# Organotypic pancreatoids with native mesenchyme develop Insulin producing endocrine cells

**DOI:** 10.1038/s41598-017-11169-1

**Published:** 2017-09-07

**Authors:** Marissa A. Scavuzzo, Diane Yang, Malgorzata Borowiak

**Affiliations:** 1 Program in Developmental Biology, Baylor College of Medicine, Houston, TX 77030 USA; 2 Molecular and Cellular Biology Department, Baylor College of Medicine, Houston, TX 77030 USA; 3 Center for Cell and Gene Therapy, Baylor College of Medicine, Texas Children’s Hospital, and Houston Methodist Hospital, Houston, TX 77030 USA; 4 Stem Cell and Regenerative Medicine Center, Baylor College of Medicine, Houston, TX 77030 USA; 5 McNair Medical Institute, Baylor College of Medicine, Houston, TX 77030 USA

## Abstract

Replacement of lost beta cells in patients with diabetes has the potential to alleviate them of their disease, yet current protocols to make beta cells are inadequate for therapy. *In vitro* screens can reveal the signals necessary for endocrine maturation to improve beta cell production, however the complexities of *in vivo* development that lead to beta cell formation are lost in two-dimensional systems. Here, we create three-dimensional organotypic pancreatic cultures, named pancreatoids, composed of embryonic day 10.5 murine epithelial progenitors and native mesenchyme. These progenitors assemble in scaffold-free, floating conditions and, with the inclusion of native mesenchyme, develop into pancreatoids expressing markers of different pancreatic lineages including endocrine-like cells. Treatment of pancreatoids with (−)-Indolactam-V or phorbol 12-myristate 13-acetate, two protein kinase C activators, leads to altered morphology which otherwise would be overlooked in two-dimensional systems. Protein kinase C activation also led to fewer Insulin+ cells, decreased *Ins1* and *Ins2* mRNA levels, and increased *Pdx1* and *Hes1* mRNA levels with a high number of DBA+ cells. Thus, organotypic pancreatoids provide a useful tool for developmental studies, and can further be used for disease modeling, small molecules and genetic screens, or applied to human pluripotent stem cell differentiation for beta-like cell formation.

## Introduction

The pancreas, nestled between the stomach and the intestine, is a physiological juggernaut responsible for regulating digestion and blood glucose homeostasis. These physiological feats are achieved through the coordinated functions of diverse cell types: acinar cells secrete enzymes into a pancreatic ductal system that empties into the duodenum to break down food, while four different endocrine cell types release different hormones to finely calibrate blood glucose levels and feedback on digestive activities. Gaining an understanding of mechanisms governing pancreatic development will not only improve our understanding of pancreatic diseases, but also advance cell-based therapies, which hinge upon mimicking *in vivo* developmental processes in an *in vitro* context. These cell-based therapies are particularly pressing for diabetes, which is characterized by a loss or dysfunction of Insulin producing endocrine beta cells, leaving patients hyperglycemic and affecting 415 million people worldwide. Replacing these cells has potential to render patients asymptomatic, yet our knowledge regarding pancreatic development is insufficient to make fully functional beta cells on a large enough scale for clinical impact. Studies in mouse models have provided a wealth of information that can then be applied to human stem cell differentiation^1–3^, however *in vivo* manipulation of the mouse pancreas during embryogenesis through current methods is time consuming and labor intensive. Use of cultured cells, while beneficial for screening purposes, loses the three-dimensional architecture, cellular interactions, and cellular diversity present in *in vivo* development. Thus it is essential for the derivation of new model systems that can 1) maintain the complexity of the native developing pancreas, 2) allow analysis of early pancreatic embryogenesis and fate determination, and 3) be applicable for screening purposes.

Pancreatic embryogenesis can be divided into two phases. During the primary transition (mouse e8.5-e12.5), highly proliferative multipotent pancreatic progenitors are specified from the gut tube and bud out, before the cells undergo fate restrictions and traverse through different developmental routes to differentiate during the secondary transition (mouse e12.5-e17.5). The mesenchyme that surrounds the developing pancreatic epithelium aids in progenitor expansion and subsequent differentiation^4–8^. In fact, when endocrine cells are induced from the epithelium in the secondary transition, they delaminate and migrate across the mesenchyme before differentiating into mature hormone producing endocrine cells^9^. Studies have further shown that co-culture with mesenchyme or treatment with factors derived from mesenchyme increases beta cell formation *in vitro* ^10, 11^, illustrating the importance of these tissue interactions during pancreatic embryogenesis.

Recent advances by Greggio, *et al*.^12^ have led to the establishment of a three-dimensional pancreatic murine organoid culture system, in which multipotent progenitors were isolated from the e10.5 mouse pancreatic bud, mesenchymal tissue was completely removed, and cells were cultured in Matrigel scaffolds in carefully constructed media to promote their organization and differentiation. This, for the first time, marks the creation of pancreatic organoids that acquire the cellular complexity, organization, and morphology reminiscent of the inherent murine pancreas. However, a scarce number of endocrine cells (0.17%) develop in the three-dimensional Matrigel culture. When these organoids were grafted into the e13.5 pancreas, the pancreatic niche, where mesenchyme is abundant, supported the differentiation of 2–5% of organoid progenitors into endocrine cells. The recovery of endocrine differentiation with engraftment shows the necessity of the niche in fostering proper development into all of the pancreatic lineages including endocrine cells. Here, we developed scaffold-free organotypic three-dimensional pancreatic cultures (pancreatoids) that retain their native mesenchyme and effectively develop into all of the lineages of the pancreas, including Insulin producing endocrine-like cells (22.42%). This allows not only analysis of mechanisms dictating endocrine development and disease, but exocrine cells as well. These organotypic pancreatoids can be used as a tool to study signaling pathways involved in pancreatic development or disease. Using this model, we analyze the effects of protein kinase C (PKC) activation using different small molecules, (−)-Indolactam-V (ILV) and phorbol 12-myristate 13-acetate (PMA) on early stages of pancreatic development and morphology. Thus scaffold-free organotypic pancreatoids impart a valuable resource for analysis of pancreatic development.

## Results

### Scaffold-free organotypic three-dimensional cultures of pancreatic epithelium and native mesenchyme

**Figure 1:**
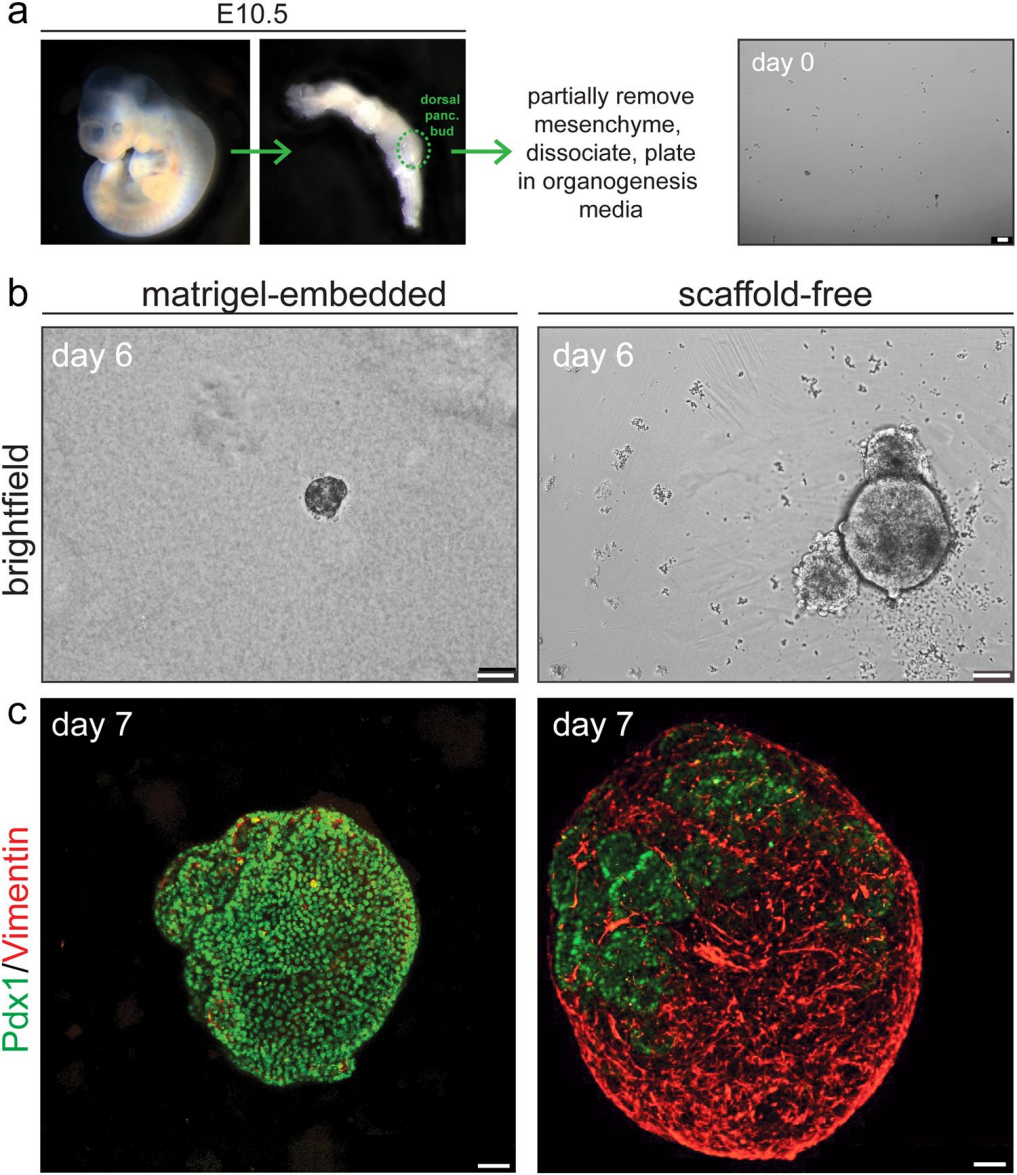
Residual mesenchyme envelops pancreatic epithelium in scaffold-free, three-dimensional organotypic culture. (**a**) Schematic procedure for the generation of organotypic pancreatoids. Dissection of the dorsal pancreatic bud (dorsal panc. bud) at embryonic day 10.5 (E10.5) precedes removal of mesenchyme by dispase and dissociation of cells before seeding in organogenesis media. Scale bar = 50 um. (**b**) Brightfield images of Matrigel-embedded organoids with mesenchyme removed and scaffold-free, free-floating organotypic pancreatoids. Scale bar = 50 um. (**c**) Whole mount three-dimensional reconstruction of Matrigel-embedded organoid and scaffold-free organotypic pancreatoid stained by immunofluorescence, with the epithelium marked by Pdx1 (in green) and mesenchyme marked by Vimentin (in red). Scale bar = 50 um.

To generate pancreatoids, we dissected the dorsal pancreatic bud from e10.5 mouse gut tube when cells are multipotent^13, 14^ before dissociating cells and culturing in organogenesis media (Fig. 1a; Greggio, *et al*.^12^). We reproduced the pancreatic organoids devoid of mesenchyme embedded in a Matrigel scaffold as described by Greggio, *et al*.^12^ prior to experimentation of culture conditions. As a means to determine the contribution of the native mesenchyme to pancreatic embryogenesis but prevent mesenchymal overgrowth, we reduced the amount of mesenchyme through use of dispase but without further mechanical removal. The removal of the majority of mesenchyme by dissecting the bud away from the surrounding niche before incubating in dispase was essential to prevent over proliferation of mesenchyme, which at higher amounts out competed the other tissue and impeded pancreatoid development (data not shown). At day 0, after partial removal of the mesenchyme, dissociated cells were plated free floating in ultra low attachment plates at a 1-to-4, bud-to-pancreatoid ratio (Fig. 1a).

At day 6, Matrigel-embedded pancreatic organoids and scaffold-free, floating organotypic pancreatoids were similar in structure (Fig. 1b), although the pancreatoids were slightly larger in size (4.4-fold ± 1.6, n = 6, *p* = 0.01 by unpaired Student’s t-test). To determine the disparity in the quantity of mesenchyme remaining between protocols, we immunostained organoids and pancreatoids at day 7 and imaged in three-dimensions. Matrigel-embedded pancreatic organoids had minimal mesenchyme, as marked by Vimentin in red, showing the efficiency of chemical (dispase) and mechanical (using needles) removal, leaving only epithelial cells marked by Pdx1 in green. In contrast, organotypic pancreatoids were heavily enveloped in mesenchyme, showing that even after removal with dispase the mesenchyme recurs (Fig. 1c). The pancreatoids were composed on average of 24,800 cells after 7 days in culture (n = 5, SEM = 4363.48). Further, we found that organotypic pancreatoids formed even from the e11.5 mouse dorsal pancreas, showing that these cells were still multipotent or plastic (Supplemental Fig. 1). Overall, we established new protocol to generate scaffold-free, floating organotypic pancreatoids that retain the native mesenchyme to foster epithelial development.

### Formation of exocrine-like and endocrine-like cells in organotypic pancreatoids

**Figure 2:**
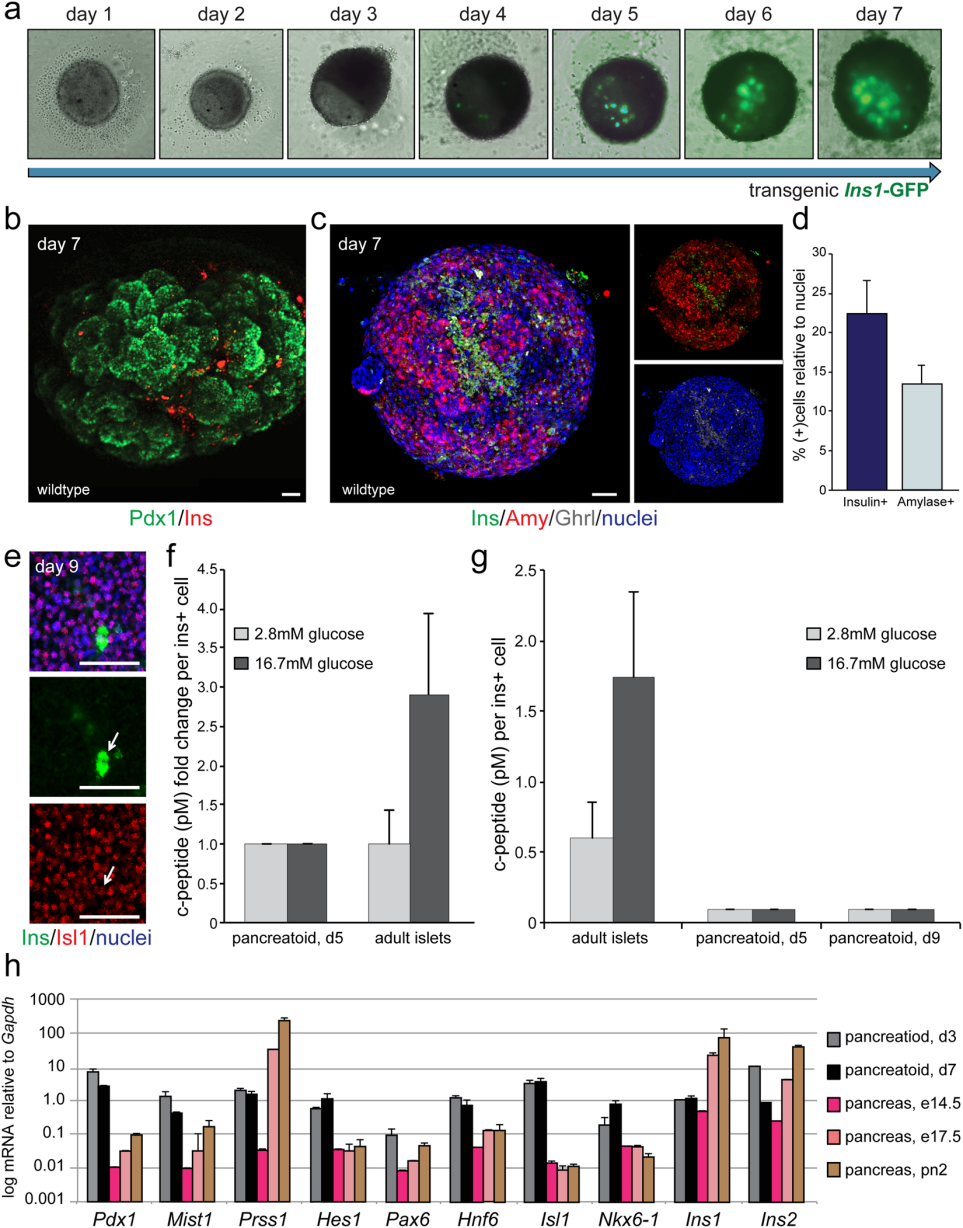
Organotypic pancreatoids develop Insulin producing beta cells. (**a**) Time course of organotypic pancreatoid development from *Insulin1*- EGFP mice. (**b**) Whole mount three-dimensional reconstruction of organotypic pancreatoid from a wildtype mouse at day 7 stained by immunofluorescence, with the epithelium marked by Pdx1 (in green) and beta-like cells marked by Ins (in red). Scale bar = 50 um. (**c**) Whole mount three-dimensional reconstruction of organotypic pancreatoid from a wildtype mouse at day 7 stained by immunofluorescence, with the acinar-like cells marked by Amy (in red), beta-like cells marked by Ins (in green), and endocrine progenitors marked by Ghrl (in white). Nuclei are marked by dapi in blue. Scale bar = 50 um. (**d**) Quantification of acinar-like and beta-like cell proportions. N = 8. Error bars are SEM. (**e**) Whole mount three-dimensional reconstruction of organotypic pancreatoid from a wildtype mouse at day 7 stained by immunofluorescence, with the beta-like cells marked by Ins (in green) and endocrine progenitors marked by Isl1 (in red). Nuclei are marked by dapi in blue. Scale bar = 50 um. (**f**) Fold change relative to 2.8 mM glucose showing c-peptide secretion during GSIS normalized to Ins+ cell number. N = 3. Error bars are SEM. (**g**) Absolute values of c-peptide secretion during GSIS normalized to Ins+ cell number. N = 3. Error bars are SEM. (**h**) Quantitative PCR to determine gene expression of key pancreatic markers, including *Pdx1* (epithelium), *Mist1*, *Prss1*, and *Hes1* (exocrine), and *Pax6*, *Hnf6*, *Isl1*, *Nkx6-1*, *Ins1*, and *Ins2* (endocrine). Y-axis scale is log10. Expression is normalized to *Gapdh*. N = 4 for pancreatoids, N = 3 for murine tissue. Error bars are SEM.

The embryonic pancreatic mesenchyme influences the differentiation of all epithelial pancreatic cell types^6–8^. Greggio, *et al*.^12^ observed a paucity of endocrine cells in Matrigel-embedded organoids (0.17%), but an increased number of endocrine cells (2–5%) after embedding into the e13.5 pancreas, where the progenitors can interact with the native niche at a critical developmental stage of the secondary transition. This led us to hypothesize that the organotypic pancreatoids with residual mesenchyme may have an improved ability to form endocrine cells, as the niche is still present to provide signals to instruct differentiation. To test this, we generated pancreatoids from mice expressing EGFP under the control of the Insulin-1 promoter to visualize the formation of Insulin+ cells during a time course^15^. The organotypic pancreatoids began to show Insulin expression at day 4, which increased in number until day 7 (Fig. 2a–c; Supplemental Fig. 2). Insulin expression was also observed at day 7 of wildtype pancreatoids, while a high number of cells expressed Pdx1 (Fig. 2b). Pancreatoids also developed Amylase+ cells, which did not overlap with Insulin expression, with cells expressing the two markers segregating and clustering together (Fig. 2c). While the larger pancreatoids developed in closer proportions to the pancreas *in vivo*, the majority of pancreatoids were composed of a high percentage of Insulin+ cells (22.4%) and fewer Amylase+ cells (13.4%, Fig. 2d). Further, we observed Ghrelin expression in all of the Insulin+ cells but no Amylase+ cells, along with Isl1 expression (Fig. 2c,e). Ghrelin has been reported to be expressed in embryonic pancreatic progenitors before development into endocrine cells^16^, thus these Insulin+ cells may be immature or fetal-like.

To gain insight into the functionality of the of these pancreatoid-derived beta-like cells, we performed glucose stimulated insulin secretion assays and measured the level of c-peptide from pancreatoids at day 5, day 9, and of isolated adult mouse islets. We find that while pancreatoids secrete a small level of c-peptide (0.09pM/cell), they do not increase this secretion after high glucose stimulation (16.7 mM) but remain at basal levels (Fig. 2f). Pancreatoids also do not gain glucose-responsiveness after a longer time in culture, with day 9 pancreatoids almost identical in c-peptide levels and secretion after stimulation as day 5 pancreatoids (Fig. 2g). Thus the beta-like cells developing and expressing Insulin in the pancreatoids are not mature, glucose responsive beta cells. To determine what stage of pancreatic development the pancreatoids most closely resemble in gene expression, we performed a qPCR screen of different endocrine and exocrine markers (Fig. 2h). Pancreatoids at day 3 and day 7 had high expression of *Pdx1*, which may reflect a difference in the ratio of mesenchyme to epithelium in pancreatoids compared to the embryonic and early postnatal pancreas. A higher level of *Mist1, Hes1, Hnf6, Isl1*, and *Nkx6-1* was observed in both d3 and d7 pancreatoids compared to all *in vivo* tissue stages analyzed, while *Prss1, Pax6*, *Ins1*, and *Ins2* more closely resembled e17.5 and postnatal day 2 pancreatic tissue (qPCR primers listed in Table 1).

**Table 1:** qPCR primers.

Gene	f (5′–3′)	r (5′–3′)
Gapdh	AATGTGTCCGTCGTGGATCTGA	AGTGTAGCCCAAGATGCCCTTC
Nkx6.1	GACCTGACTCTCCGTCATCC	TTGTTGGACAAAGATGGGAAG
Pax6	TCTAATCGAAGGGCCAAATG	AGGAGGAGACAGGTGTGGTG
Hnf6	TTCTTGCTCTTTCCGTTTGC	CCCTGGAGCAAACTCAAGTC
Ins1	GGAGCGTGGCTTCTTCTACA	CTGCAGCACTGATCCACAAT
Ins2	TCTACAATGCCACCACGCTTCTG	TTTGTCAAGCAGCACCTTTG
Prss1	GTGGTAGCCAGAGTTCAGGG	ACTCCTGTTCCTGGCCCTT
Isl1	GCATTTGATCCCGTACAACC	CTGAGGGTTTCTCCGGATTT
Mist1	TCATAGCTCCAGGCTGGTTT	AAGCTACGTGTCCTTGTCCC
Pdx1	CCACCCCAGTTTACAAGCTC	CGTGAGCTTTGGTGGATTTC
Hes1	CCAAGCTAGAGAAGGCAGACA	TGATCTGGGTCATGCAGTTG

**Figure 3:**
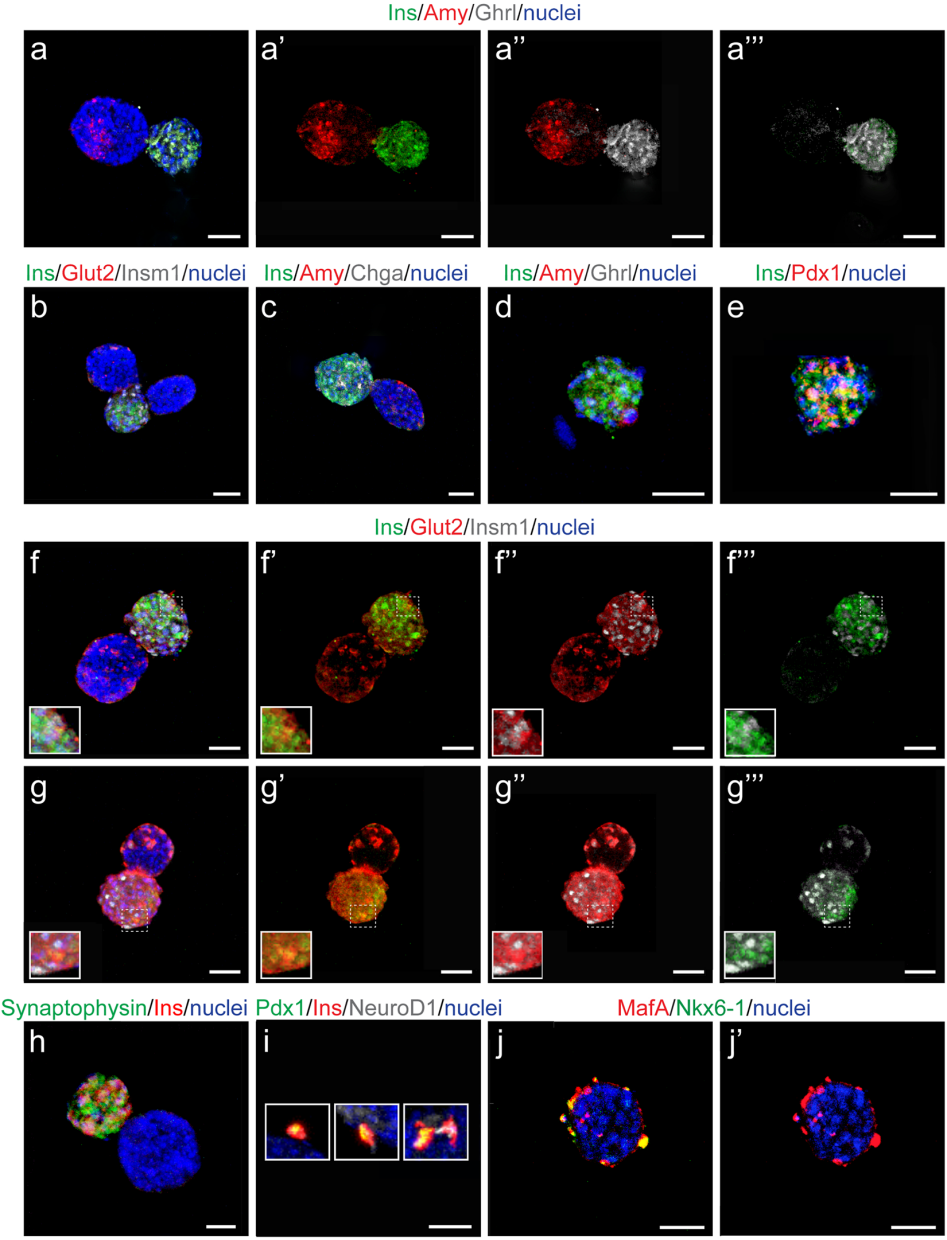
Pancreatoid endocrine-like cells express beta cell markers. (**a**) Three-dimensional immunofluorescence with beta-like cells marked by Ins (in green), acinar-like cells marked by Amy (in red), and endocrine progenitors marked by Ghrl (in white). Separate channels show segregation of Amy/Ins (a’), Amy/Ghrl (a”), and co-expression of Ins/Ghrl (a”’). (**b**) Three-dimensional immunofluorescence with beta-like cells marked by Ins (in green), the glucose transporter Glut2 (in red), and endocrine progenitors marked by Insm1 (in white). (**c**) Three-dimensional immunofluorescence with beta-like cells marked by Ins (in green), acinar-like cells marked by Amy (in red), and endocrine progenitors marked by Chga (in white). (**d**) Three-dimensional immunofluorescence with beta-like cells marked by Ins (in green), acinar-like cells marked by Amy (in red), and endocrine progenitors marked by Ghrl (in white). (**e**) Three-dimensional immunofluorescence with beta-like cells marked by Ins (in green) with beta cell marker Pdx1 (in red). (**f**–**g**”’) Three-dimensional immunofluorescence with beta-like cells marked by Ins (in green), the glucose transporter Glut2 (in red), and endocrine progenitors marked by Insm1 (in white). (**h**) Three-dimensional immunofluorescence with beta-like cells marked by Ins (in red) and the vesicle protein marker Synaptophysin (in green). (**i**) Three-dimensional immunofluorescence with beta-like cells marked by Ins (in red) with two beta cell markers (Pdx1 in green and NeuroD1 in white). ﻿(**j-j'**)﻿ Nkx6-1 (in green) and MafA (in red). Whole mount three-dimensional reconstruction of organotypic pancreatoids from wildtype mice at day 7. Nuclei are marked by dapi in blue. Scale bar = 50 um.

As we found a high number of Insulin+ cells and fewer Amylase+ cells in d7 pancreatoids (Fig. 2d), the discrepancy in gene expression between pancreatoids and *in vivo* tissue is likely due to a difference in cellular proportions. However, as we find that Insulin+ cells are not glucose responsive, it is also possible that there are changes in gene expression levels at a cellular level. To further investigate this, we immunostained pancreatoids for a number of endocrine markers (Fig. 3). We found that a high number of budding pancreatoids composed of two similarly sized cellular masses developed, with Amylase+ cells typically segregated to one bud while Insulin+ cells remained in a separate bud (Fig. 3a,a’). This shows that pancreatoids self-organize, with acinar-like cells clustering together and away from beta-like cells. We again observed Ghrelin expression in all of the Insulin+ cells but not in the Amylase+ cells (Fig. 3a”,a”’). These Insulin+ beta-like cells also expressed Glut2, the glucose transporter expressed in mature beta cells (Fig. 3b,f,g”’), Insm1, a downstream target of the pro-endocrine gene Neurogenin3 required for endocrine formation and present in beta cells (Fig. 3b,f,g”’), and Chga, which promotes endocrine secretory granule formation (Fig. 3c)^17–19^. Pancreatoids that did not bud into multiple cellular masses but remained small still maintained patterning of Amylase+ and Insulin+ cells, with expression mutually exclusive, albeit the majority of small, singular pancreatoids expressed almost exclusively Insulin or exclusively Amylase (Fig. 3d). The beta-like cells developing co-expressed other beta cell markers with Insulin, such as the transcription factors Pdx1 and NeuroD1 (Fig. 3e,i), the vesicle protein Synaptophysin (Fig. 3h), and sparsely expressed markers of more mature beta cells such as Nkx6-1 and MafA (Fig. 3j,j’). While the beta-like cells developing *in vitro* in pancreatoids are not yet glucose responsive, they express many endocrine and beta cell markers.

**Figure 4:**
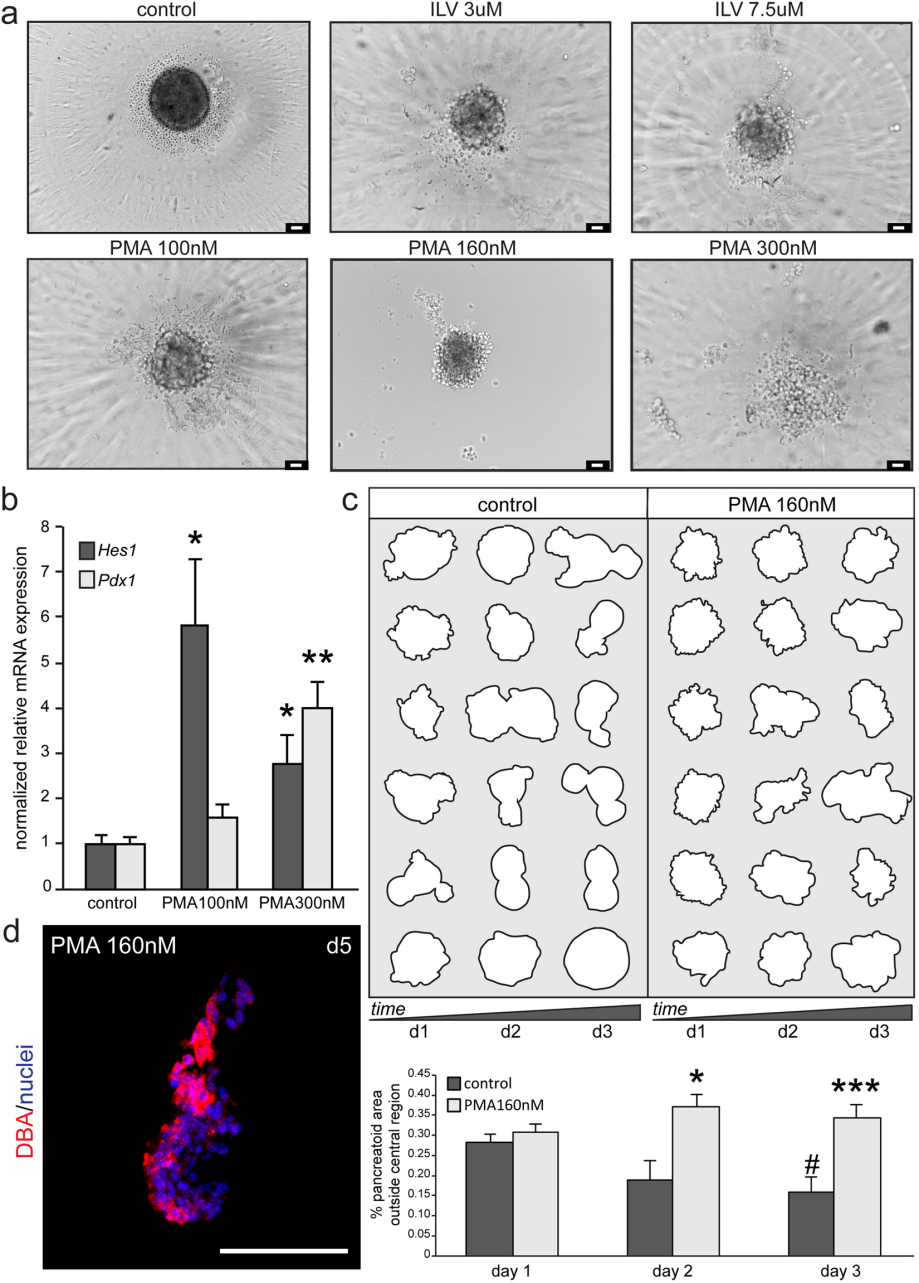
Activation of Protein Kinase C alters pancreatoid morphology. (**a**) Brightfield images of organotypic pancreatoids at day 1 of culture, in either organogenesis media (control), or organogenesis media supplemented with 3 uM or 7.5 uM of ILV, or 100 nM, 160 nM, or 300 nM of PMA. Scale bar = 50 um. (**b**) Quantitative PCR showing expression of *Pdx1* and *Hes1*. Expression is normalized to *Gapdh* and relative to control. N = 6 control and PMA 300 nM, N = 7 PMA 100 nM. Error bars are SEM. (**c**) Tracings of control and PMA 160 nM treated pancreatoids. Quantification of area outside of central region shown below. N = 6. (**d**) Three-dimensional immunofluorescence with duct-like cells marked by DBA (in red). Nuclei mark dapi in blue. Scale bar = 50 um. Data are represented as mean+ SEM (*p < 0.05, **p < 01, ***p < 0.005 control vs. PMA treated; ^#^p < 0.05 day 1 control vs. d3 control by unpaired Student’s t test).

Organotypic pancreatoids developed in this scaffold-free system regardless of the culture plate design, however those cultured in flat bottom or U bottom, ultra low attachment plates were less compact than those cultured in round, ultra low attachment spheroid microplates (Supplemental Fig. 3a). In addition, while culturing in round, ultra low attachment spheroid microplates made changing media easier due to the shape of the plate and the pancreatoid settling to the bottom in a divot, the organotypic pancreatoids formed in a shape reminiscent of the plate (Supplemental Fig. 3a,c). The spiral pattern of the plate surface is apparent in the tissue morphology as observed at day 9, by staining for Pdx1, Insulin, and Vimentin (Supplemental Fig. 3c,d). These organotypic pancreatoids still retained residual mesenchyme, which grew over the surface of the tissue, and thus developed Insulin+ cells. However, the organoids became compact in structure, lacking the morphometry of pancreatoids developed in other low attachment plates (Supplemental Fig. 3b; compared to controls in Fig. 4c). When dissociated cells from e10.5 or e11.5 were seeded into cultures with polystyrene scaffolds, the progenitors formed organotypic pancreatoids bound to the scaffolds but morphogenesis and development was not affected (Supplemental Fig. 4a,b). As these scaffolds were artificial, we then decellularized the e15.5 and adult pancreas and injected the dissociated e10.5 cells into the tissue-derived scaffolds. The progenitors formed organoids either inside the scaffolds (e15.5, Supplemental Fig. 4c), or floating near the scaffolds (adult, data not shown), but the morphology of the pancreatoids was not affected by the structure of the scaffolds and expression of different lineage markers (Insulin for beta cells, Pdx1 for epithelial cells, Amylase for acinar cells, Glucagon for alpha cells) were repressed. This supports that the native mesenchyme is important to provide signals to the epithelium to instruct differentiation, rather than to provide structural support. Thus the scaffold-free, organotypic pancreatoids developed exocrine-like and endocrine-like cells illustrating the importance of signals from the mesenchyme for pancreatic development.

### Analysis of organotypic pancreatoid morphology after activation of PKC

The generation of organotypic pancreatoids, which develop in three-dimensions, retain the complexity of the native developing pancreas, and form endocrine-like and exocrine-like cells, can be used for screening and analysis of early pancreatic embryogenesis and fate determination. Activation of Protein Kinase C (PKC) in human pluripotent stem cell (hPSC) derived definitive endoderm increases the formation of Pdx1+ pancreatic progenitors^20^, while activation of PKC at a later stage in hPSC-derived pancreatic progenitors can increase the generation of Nkx6-1+ bipotent duct/endocrine progenitors^21, 22^. We tested the outcome of increased PKC activation in organotypic pancreatoids by plating dissociated cells in organogenesis media supplemented with different concentrations of two known PKC activators, (−)-Indolactam-V (ILV, 3 uM and 7.5 uM)^20, 23, 24^ and phorbol 12-myristate 13-acetate (PMA, 100 nM, 160 nM, and 300 nM)^25–27^. As the concentration of ILV and PMA increased, the morphology of the pancreatoids changed with a reciprocal increase in branching and gradually more loosely associated epithelial cells as soon as 1 day after seeding (Fig. 4a,c), with cells at the highest concentration of PMA (300 nM) barely associated. The pancreaotoids exhibited an increase in *Pdx1* and *Hes1* expression after activation of PKC at day 3 (Fig. 4b, Table 1). As the pancreas develops through the secondary transition, proximal epithelial trunks composed of bipotent progenitors increase expression of *Hes1* and *Pdx1* ^28–30^, thus this shift in expression may indicate the branching phenotype either originates from a higher formation of bipotent progenitors or retention of cells in the progenitor state^31–33^. We indeed found a high expression of DBA, a marker of pancreatic ducts, in PMA treated pancreatoids at day 5 (Fig. 4d). Using this system as a model for pancreatic development, questions regarding pancreatic morphology, tissue architecture, and complex cellular interactions can be addressed.

### Decreased beta cell formation in PKC stimulated organotypic pancreatoids

**Figure 5:**
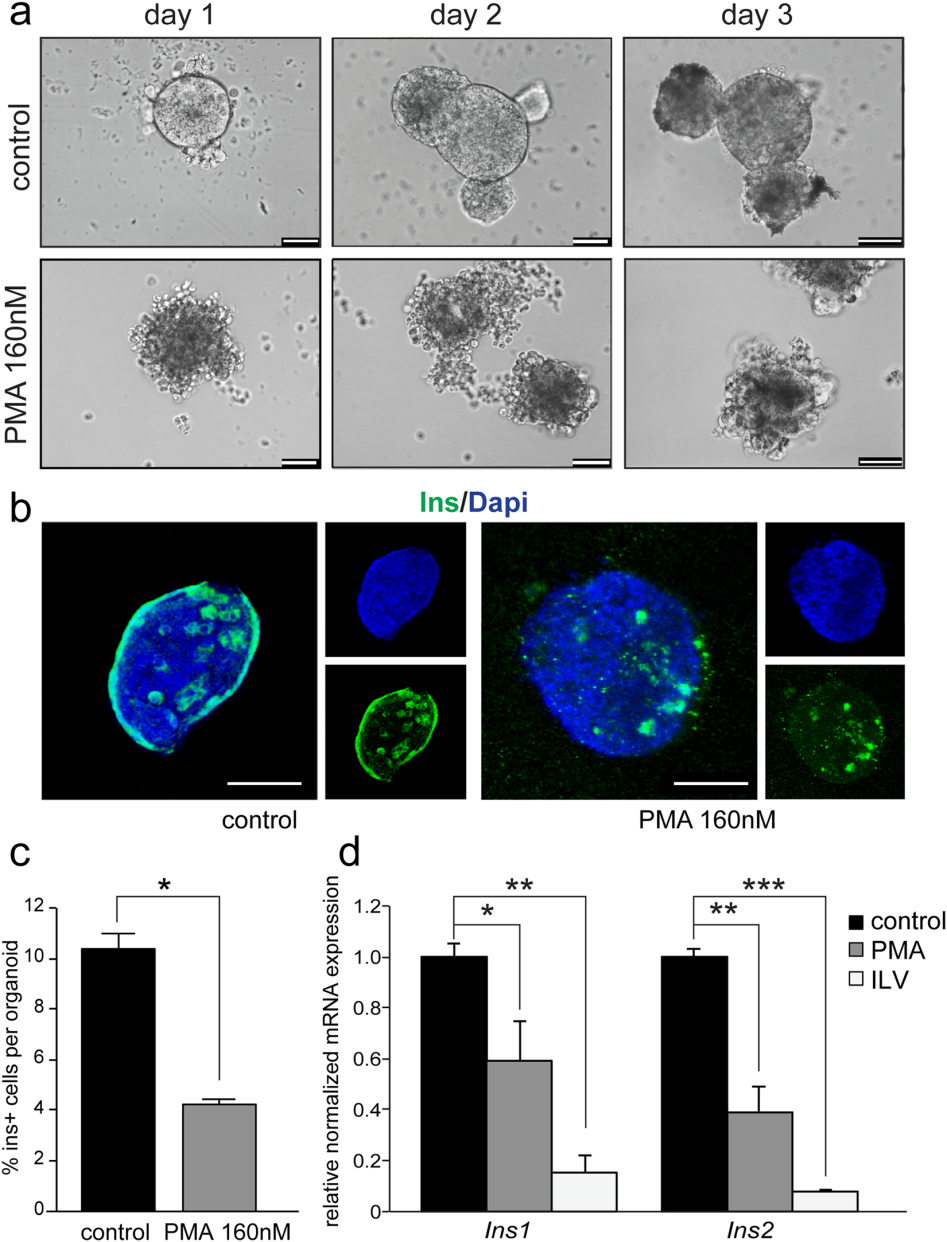
Protein Kinase C activation reduces the formation of Insulin producing cells in organotypic pancreatoids. (**a**) Brightfield images of organotypic pancreatoids at day 1, day 2, and day 3 of culture, in either organogenesis media (control), or organogenesis media supplemented with 160 nm of PMA. Scale bar = 50 um. (**b**) Whole mount three-dimensional reconstruction of organotypic pancreatoid at day 9 stained by immunofluorescence, with endocrine beta cells marked by Ins (in green) and nuclei marked by Dapi (in blue). Scale bar = 50 um. (**c**) Quantification of the percentage of Insulin+ cell number compared to total nuclei number (Dapi+) from immunofluorescent stainings. Only Insulin+ cells with clear Dapi+ were included in the analysis, with peripheral staining in the control excluded. Error bars are SEM. N = 3. *P* < 0.05. (**d**) Quantitative PCR of *Ins1* and *Ins2* in control, PMA, and ILV treated pancreatoids at day3. Expression is normalized to *Gapdh*. N = 3 control, N = 5 PMA, and N = 3 ILV. Data are represented as mean+ SEM (*p < 0.05, **p < 01, ***p < 0.005 by unpaired Student’s t test).

As development proceeded from day 1 to day 3 in organotypic pancreatoids with activated PKC, cells become more compact (Figs 4a,c and 5a). As pancreatic morphology is tightly linked with the formation of endocrine cells^34, 35^, we next determined the effect of PKC activation on beta-like cell formation. Pancreatoids either cultured for 9 days in normal organogenesis media or organogenesis media supplemented with 160 nM PMA were immunostained and imaged in three-dimensions (Fig. 5b). Pancreatoids in normal organogenesis media developed a higher percentage of Insulin+ cells compared to PMA treated pancreatoids (Fig. 5c), which was further supported by a decrease in *Ins1* and *Ins2* expression after 7 days with PMA or ILV treatment (Fig. 5d, Table 1). The increased *Pdx1* and *Hes1* expression but decreased Insulin+ cell number and *Ins1* and *Ins2* expression after PKC activation indicates that more bipotent progenitor-like cells are forming but fewer are differentiating into endocrine-like cells, with PKC acting in this stage to maintain the progenitor state. Activation of PKC at the pancreatic progenitor stage of scaffold-free, pancreatoids influenced morphological changes and shifts in the expression of several markers, including a decrease in the number of Insulin+ cells.

## Discussion

The establishment of a model system that preserves the three-dimensional architecture and cellular complexity of the developing pancreas but can be used for *in vitro* screening will provide a valuable tool. Past studies have greatly advanced our abilities to make pancreatic organoids^12^, yet these organoids are still not completely comparable to *in vivo* development, most notably lacking in the formation of endocrine cells. The association of pancreatic mesenchyme with the developing epithelium is important for the formation of both exocrine and endocrine cells^4–8, 10, 11, 36, 37^. The mesenchyme is known to provide critical factors, including FGF9, HGF, IGF, and PDGF and potentially other factors yet to be described^6, 10^. This led to our modification of existing methods to include the native pancreatic mesenchyme, generating organotypic pancreatoids composed of both mesenchyme and epithelial cells. These pancreatoids developed not only exocrine-like cell types but also endocrine-like cells, including Insulin-producing beta-like cells that express beta cell markers and produce c-peptide but are not glucose responsive. Further investigation into the mechanisms of beta cell maturation into functional, glucose responsive cells should be pursued and can be accomplished using this system. As this is an *in vitro* system, this can be used for functional screens of small molecules, drugs, or perform forward genetic screens using pooled shRNA or CRISPR sgRNAs for studies on development and pathogenesis. In this study we show the effects of PKC activation on pancreatoid development, with morphological changes that would be overlooked in a standard two-dimensional system. Currently there is a great effort to create glucose-responsive beta cells from hPSCs, and while the field is getting closer to achieving this goal, it has been hindered by gaps in knowledge regarding native pancreatic embryogenesis and development. Screens in hPSCs have a high monetary and temporal cost (approximately 6 weeks), while pancreatoids develop endocrine-like cells within just a few days. While there are distinctions between mouse and human islet development, knowledge regarding the mechanisms of mouse endocrine development can facilitate progress in hPSC differentiation. Finding the factors that make pancreatoids glucose responsive will provide insight into possible means to produce glucose responsive cells *in vitro* from hPSCs.

Further, organotypic pancreatoids can be used to model diseases or for drug screening through genetic or chemical perturbation. While this provides a step forward, the removal of mesenchyme must be precise to eliminate enough to prevent overgrowth but leave enough to envelop the pancreatoid. In addition, the effect of culture conditions on the mesenchyme has not yet been determined, and it is possible that *in vivo*, the mesenchyme shifts as development proceeds, providing different signals at different stages. Culturing epithelial organoids with pancreatic mesenchyme derived from different stages of embryogenesis may allow for more careful introduction of mesenchyme at specific cell numbers and stage specificity in relation to epithelium. As the localization and proportion of Ins+ cells in our pancreatoids were not identical to the endogenous pancreas, we are interested in investigating what is contributing to these differences. For instance, we did not observe a significant number of Pecam+ cells in the pancreatoids (data not shown); however endothelial cells are known to play an important role in endocrine differentiation^38^ and support beta cell physiology. In addition, neural innervation is not conserved in our system. Identification of critical factors not only from the mesenchyme but also from endothelial cells and neurons would provide valuable insight for *in vitro* differentiation, and can be applied to our pancreatoid system to more closely resemble endogenous development. We also observed roughly 23% Insulin+ cells at d7, while only 11% were Insulin+ at d9. This difference in Insulin+ cell number may be due to proliferation of other cell types in the pancreatiod, however further investigation is necessary.

As we build upon the conditions necessary to generate murine pancreatoids, we can apply this knowledge in differentiating hPSCs to work towards the development of three-dimensional, multicellular, diverse human pancreatic organoids. Given that the differentiation of hPSCs to the pancreatic progenitor stage is robust, cells grown in conventional two-dimensional protocols can be seeded free floating in non-attachment plates in organogenesis media to evaluate their ability to form organoids. Past studies have cocultured murine tissue with mesenchymal cell lines, and coculture of hPSCs differentiating to the pancreatic lineage with stage matched mesenchyme greatly increases the expansion of progenitors and their subsequent differentiation potential^8^. Thus coculturing hPSC-derived pancreatic progenitors with mesenchymal cell lines may further improve pancreatic maturation *in vitro*, creating a platform to study new signals for endocrine maturation. Further, identification of factors from the mesenchyme that influence pancreatic differentiation and support endocrine formation will improve *in vitro* protocols as these can be applied as growth factors to differentiating cells in two- or three-dimension. The generation of human pancreatoids by applying the knowledge determined here can provide a system to study human pancreatic development, human disease, or to even grow as a tissue source for transplantation.

## Methods

### Animals

Animal studies were approved by the Baylor College of Medicine Institutional Animal Care and Use Committee and carried out in accordance to guidelines and regulations. Mice were housed at 22–24 °C with a 12 hour light/12 hour dark cycle with standard chow (Lab Diet Pico Lab 5V5R, 14.7% calories from fat, 63.3% calories from carbohydrate, 22.0% calories from protein) and water provided ad libitum. All mice studied were on a mixed background (129SvEv, FVB, and C57BL/6 J).

We used C57BL/6 mice for all experiments unless otherwise stated. Transgenic *Insulin1*-EGFP mice were obtained from (Jax Laboratories, #006864). E0.5 was defined as noon the day the vaginal plug was observed in the mother.

### Murine pancreatic organogenesis protocol

The dorsal pancreatic bud was dissected from embryonic day 10.5 embryos and prepared as described by Greggio *et al*. (2013) for Matrigel embedded organoids. To prepare scaffold free organoids, e10.5 dorsal pancreas was isolated and placed in PBS in a 12-well plate on ice until all buds were collected. Buds were transferred using a flame pulled capillary attached to a mouth pipette to 1.25 mg/mL cold dispase for 2–3 minutes before rinsing in PBS twice. Unless large portions of mesenchyme were visible, the mesenchyme was not further dissociated at this point to allow a small amount of residual mesenchyme to remain. Buds were collected in a LoBind Protein microcentrifuge tube (Eppendorf) and suspended in 1 mL of 0.05% trypsin (Gibco) at 37 °C for 5 minutes. Cells were dissociated by briefly vortexing before spinning down at 5000 g for 5 minutes. Trypsin was removed and cells were resuspended in at least 200 uL of organogenesis media per bud, and plated at 50 uL in either cell repellent round, ultra low attachment spheroid microplates (Corning 4520) or in 500 uL in 24 well flat bottom ultra low attachment plates (Corning 3473), splitting buds at a 1:4 ratio. Medium was replaced every 3–4 days and the growing pancreatic organoids were monitored daily.

The organogenesis medium was composed as described by Greggio *et al*. (2013), as follows: DMEM/Nutrient Mixture F12 (DMEM/F12; Gibco); 1% penicillin-streptomycin; 10% KnockOut Serum Replacement (KSR; Gibco); 0.1 mM 2-mercaptoethanol; 16 nM phorbol 12-myristate 13-acetate (PMA; Calbiochem); 10 uM Y-27632 (ROCK inhibitor; Sigma); 25 ng/mL EGF (Sigma); 500 ng/mL mouse R-spondin 1 (R&D Systems); 100 ng/mL FGF10 (R&D Systems); 25 ng/mL FGF1 (PeproTech); 2 U/mL heparin (Sigma).

For activation of protein kinase C, PMA concentration was raised to 100 nm, 160 nm, or 300 nm as indicated, while (−)-Indolactam V ILV (Enzo Life Sciences) was added at 3 uM or 7.5 uM.

### Microscopy

Low magnification images of e10.5 mouse and digestive tract were captured through a Leica M60 microscope. All brightfield images and monitoring of Ins1-EGFP transgene expression were taken on a Leica CTR DM6000 FS epifluorescent microscope. Confocal images (all tissue stained with immunofluorescence) were imaged using a Leica TCS SPE or Zeiss 710 microscope.

### Decellularization and polystyrene scaffolds

Tissue from e15.5 or adult pancreas was isolated, washed in PBS, and incubated with 0.1% SDS in water for 24–48 hours until the tissue appeared transparent. The tissue was then placed in water for 15 minutes before 1% Triton-X for 10 minutes, washing in PBS with 1% penicillin-streptomycin, and storing in PBS with 1% penicillin-streptomycin at 4 °C until use. Dissociated cells from e15.5 or adult pancreas were either plated in the organogenesis media with the scaffolds or suspended in organogenesis media and injected using a 26-gauge needle.

Polystyrene scaffolds were constructed by scraping the bottom of polystyrene plates with a 20-gauge needle to generate strands of polystyrene, which was plated in organogenesis media with dissociated cells.

### qRT-PCR

Total RNA was isolated by phenol-chloroform extraction using TRIzol (Thermo Fischer Scientific). DNA was depleted by DNAse treatment (Invitrogen) and then reverse transcribed using iScript cDNA Synthesis kit (BioRad). Real-time PCR was performed with diluted cDNAs in a 15uL reaction volume using Kapa SYBR Fast qPCR mix in duplicates and analyzed using the CFX96 Touch Real-Time PCR Detection System. ΔΔCq was computed using the CFX96 software. Genes were normalized to *GAPDH*. Primers were as follows:

### Insulin secretion

Organotypic pancreatoids were washed 4 times with KREBS buffer (1.26 mM NaCl, 25 mM KCl, 250 mM NaHCO_3_, 12 mM NaH_2_PO_4_, 12 mM MgCl_2_, and 25 mM CaCl_2_) and incubated for 1 hour before collection of media for c-peptide ELISA. Organotypic pancreatoids were dispersed into single cells using TrypLE Express and cell number was counted. Mouse islets were washed with Krebs buffer (1.26 mM NaCl, 25 mM KCl, 250 mM NaHCO_3_, 12 mM NaH_2_PO_4_, 12 mM MgCl_2_, and 25 mM CaCl_2_) and preincubated in Krebs buffer for 2 hours at 37 °C to remove residual insulin. Clusters were incubated in low-glucose (2.8 mM) conditions for 30 minutes before stimulation with high-glucose (16.7 mM) for 30 minutes. Clusters were then dispersed into single cells using TrypLE Express and cell number was counted. C-peptide content was determined using Mouse c-peptide ELISA kit (ALPCO Diagnostics).

### Immunofluorescence

Organotypic pancreatoids were washed in PBS and fixed in 4% paraformaldehyde for 20 minutes and washed in PBS three times prior to staining or agarose embedding. Tissues were either stained whole mount or embedded into 2% agarose. Tissues were then were blocked for 2 hours room temperature with PBS+ 0.1% Triton X-100 (PBST) with 5% donkey serum (Jackson ImmunoResearch) then incubated overnight at 4 °C in primary antibodies. Tissues were washed in PBST and incubated with secondary antibody overnight at 4 °C. All secondary antibodies were purchased from Jackson ImmunoResearch. Tissues were then washed in PBST before nuclei were stained with Dapi (Invitrogen). Quantification of Insulin+ and Dapi+ cell numbers were performed using ImageJ, with the number of insulin+ cells normalized to the number of Dapi+ cells. Statistics were performed using a Student’s paired t-test.

Antibodies were used as follows: Pdx1 (DSHB at 1:100), Ins (Dako, A056401 at 1:1000), Amy (Sigma, A8273 at 1:400), Chga (DSHB at 1:200), Insm1 (Santa Cruz Biotech. sc-271408 at 1:100), DBA (Vector Lab, RL-1032 at 1:500), Vim (EMD Millipore Corp., Ab5733 at 1:500), Ghrl (Santa Cruz Biotech. SC-10368 at 1:100), Glut2 (Millipore 071–402), Isl1 (DSHB at 1:50), NeuroD1 (Santa Cruz Biotech. Sc-1084 at 1:100), Synaptophysin (Dako M0776 at 1:100), Nkx6-1 (DSHB at 1:100), and MafA (Bethyl Lab. IHC-00352 at 1:100).

### Morphometric quantification

To determine the branching/morphology of PMA treated pancreatoids, the perimeter of 6 pancreatoids were traced in Illustrator for ImageJ for data collection. The area in pixels was measured before then measuring the area of the closest oval fit in the central region of the pancreatoid to measure the area of the main mass. For budding pancreatoids, buds were counted as the main mass if they made up more than half of the mass of any other region. The area of the central mass was then subtracted from the total pancreatoid area to determine irregularity before normalizing this value to total area for % irregularity.

## Electronic supplementary material

Supplemental Information
