# Supplementary material for: Organotypic pancreatoids with native mesenchyme develop Insulin producing endocrine cells
Source: Sci Rep. 2017 Sep 7;7:10810. doi: 10.1038/s41598-017-11169-1 (PMC5589819; doi:10.1038/s41598-017-11169-1)
Supplement: Supplementary file 1 — Supplemental Information [file 41598_2017_11169_MOESM1_ESM.pdf]

Organotypic pancreatoids with native mesenchyme develop Insulin producing endocrine cells

Marissa A. Scavuzzo[1], Diane Yang[2], and Malgorzata Borowiak[1,2,3,4*]

[1]Program in Developmental Biology, Baylor College of Medicine, Houston, TX 77030, USA

[2] Molecular and Cellular Biology Department, Baylor College of Medicine, Houston, TX 77030, USA

[3]Center for Cell and Gene Therapy, Baylor College of Medicine, Texas Children's Hospital, and Houston Methodist Hospital, Houston, TX 77030, USA; Stem Cell and Regenerative Medicine Center, Baylor College of Medicine, Houston, TX 77030, USA

[4]McNair Medical Institute, Baylor College of Medicine, Houston, TX 77030

*Corresponding author, borowiak@bcm.edu

Supplemental Information

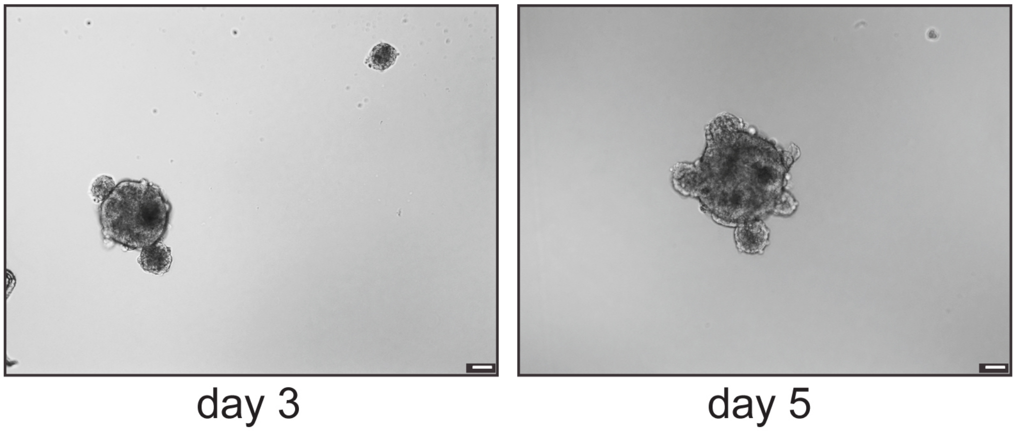

Supplemental Figure 1. Organotypic pancreatoids form from e11.5 mouse dorsal pancreatic bud. Scale bar=50um.

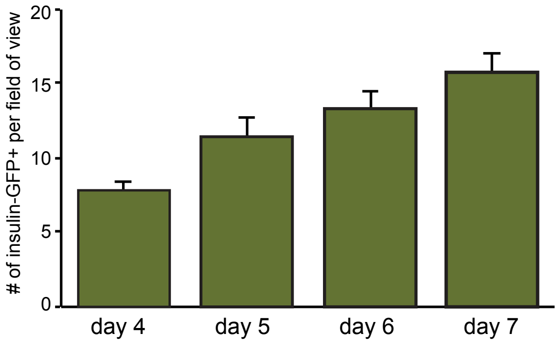

Supplemental Figure 2. Quantification of Insulin-eGFP+ cells from day 4 to day 7 as seen in Figure 2a. N=8. Error bars are SEM.

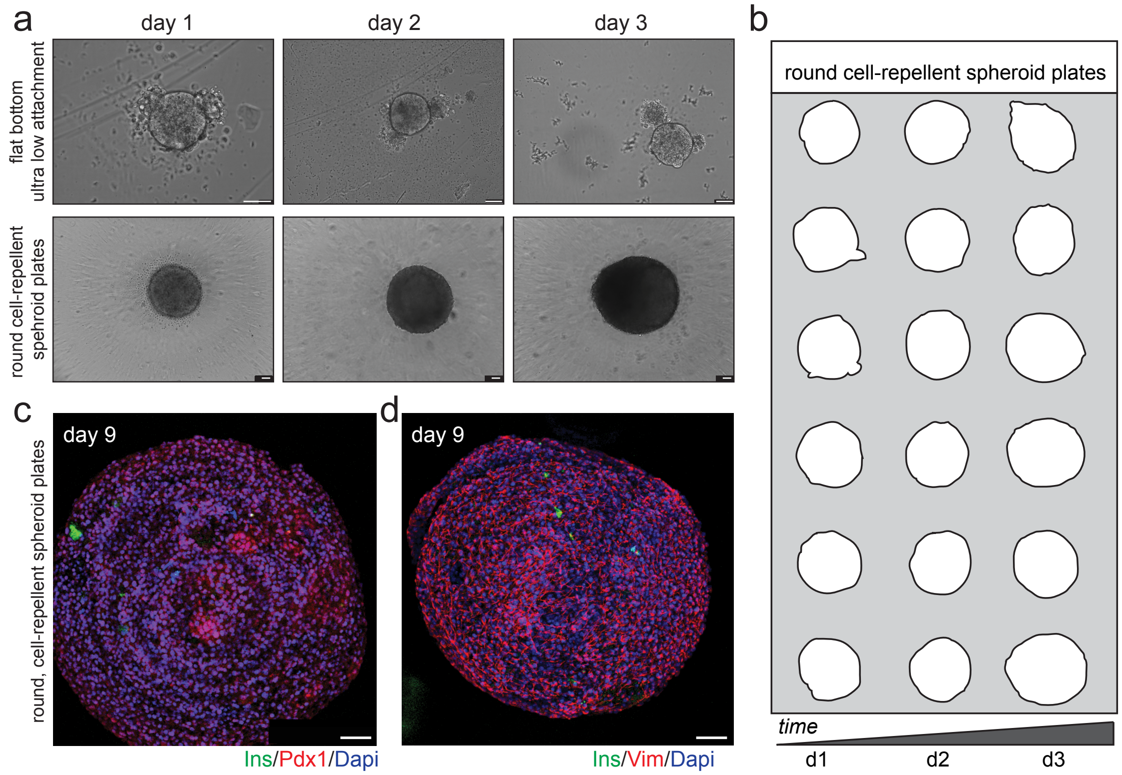

Supplemental Figure 3. Flat bottom non-attachment plates compared to round, ultra low attachment spheroid microplates for organotypic pancreatoid generation.

a) Brightfield images of organotypic pancreatoids at day 1, day 2, and day 3 in either flat bottom, non-attachment plates (top) or round, ultra low attachment spheroid microplates (bottom). Scale bar=50um.

b) Tracings of pancreatoids grown in round, cell-repellent spheroid plates over 3 days.

c) Whole mount three-dimensional reconstruction of organotypic pancreatoid at day 9 stained by immunofluorescence, with endocrine beta-like cells marked by Ins (in green) and nuclei marked by Dapi (in blue), and Pdx1

marking epithelial cells in the left panel (in red) while Vimentin marks mesenchyme in the right panel (Vim, in red). Scale bar=50um.

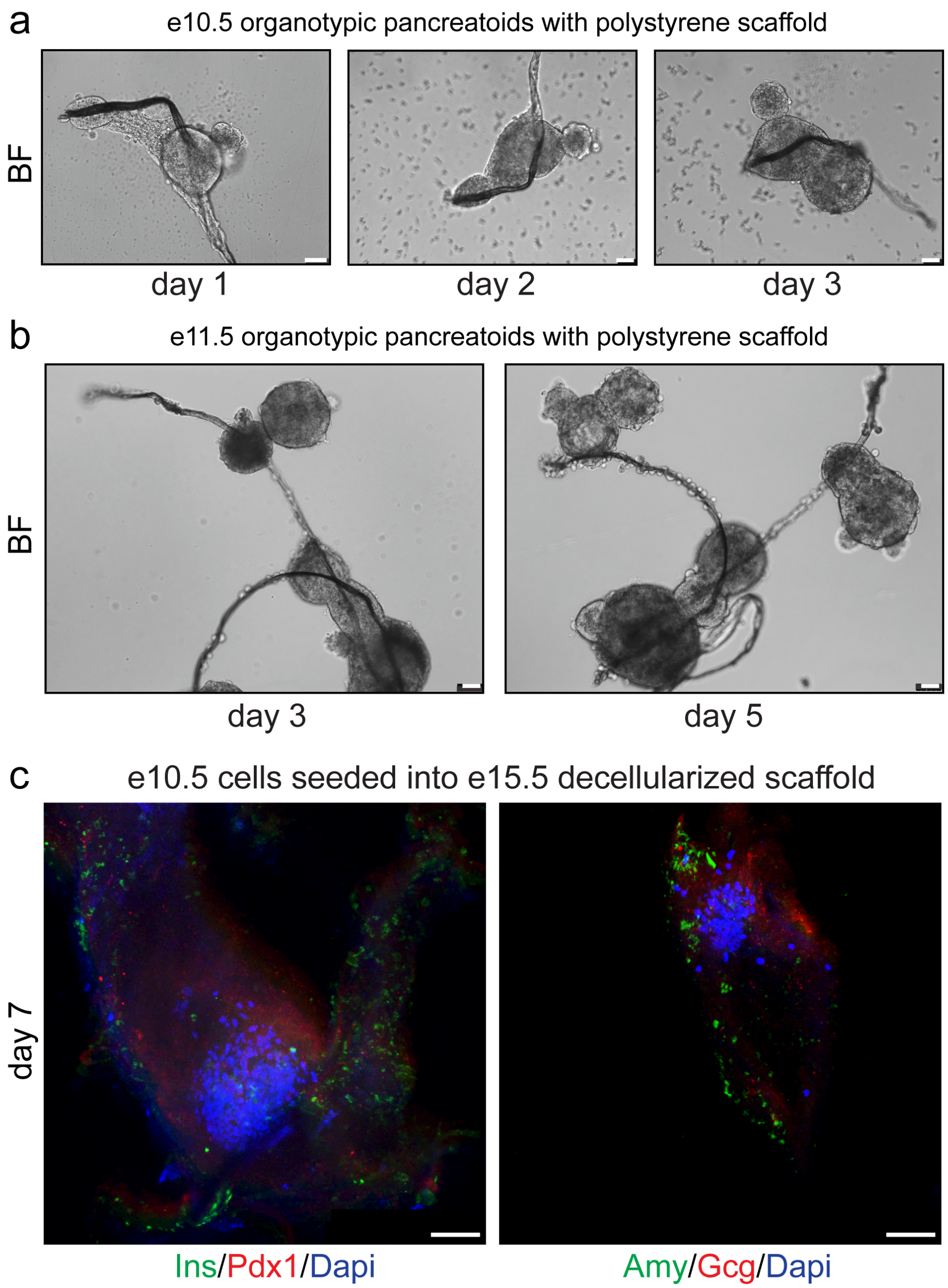

Supplemental Figure 4. Different scaffolds do not improve organotypic pancreatoid development.

a) Organotypic pancreatoids from e10.5 mouse bind to polystyrene scaffolds but still resemble free floating, scaffold free pancreatoids. Scale bar=50um.

b) Organotypic pancreatoids from e11.5 mouse bind to polystyrene scaffolds but still resemble free floating, scaffold free pancreatoids. Scale bar=50um.

c) Progenitors from e10.5 injected into decellularized e15.5 pancreatic scaffolds do not express Insulin (left panel, Ins in green), Pdx1 (left panel, in red), amylase (right panel, Amy in green), or glucagon (right panel, Gcg in red). Nuclei are marked in blue by Dapi. Scale bar=50um.
